# Analyzing the spatial and temporal distribution of human brucellosis in Azerbaijan (1995 - 2009) using spatial and spatio-temporal statistics

**DOI:** 10.1186/1471-2334-12-185

**Published:** 2012-08-08

**Authors:** Rakif Abdullayev, Ian Kracalik, Rita Ismayilova, Narmin Ustun, Ayden Talibzade, Jason K Blackburn

**Affiliations:** 1 Republican Anti-Plague Station, Baku, Azerbaijan; 2 Spatial Epidemiology and Ecology Research Laboratory, Department of Geography, University of Florida, Gainesville, FL, USA; 3 Emerging Pathogens Institute, University of Florida, Gainesville, FL, USA

**Keywords:** Brucellosis, Spatio-temporal analysis, Azerbaijan, Former Soviet Union, Serology

## Abstract

**Background:**

Human brucellosis represents a significant burden to public and veterinary health globally, including the republic of Azerbaijan. The purpose of this study was to examine and describe the spatial and temporal aspects of the epidemiology of human brucellosis in Azerbaijan from 1995 to 2009.

**Methods:**

A Geographic information system (GIS) was used to identify potential changes in the spatial and temporal distribution of human brucellosis in Azerbaijan during the study period. Epidemiological information on the age, gender, date, and location of incident cases were obtained from disease registries housed at the Republican Anti-Plague station in Baku. Cumulative incidences per 100,000 populations were calculated at the district level for three, 5-year periods. Spatial and temporal cluster analyses were performed using the Local Moran’s *I* and the Ederer-Myer-Mantel (EMM) test.

**Results:**

A total of 7,983 cases of human brucellosis were reported during the 15-year study period. Statistically significant spatial clusters were identified in each of three, five year time periods with cumulative incidence rates ranging from 101.1 (95% CI: 82.8, 124.3) to 203.0 (95% CI; 176.4, 234.8). Spatial clustering was predominant in the west early in the study during period 1 and then in the east during periods 2 and 3. The EMM test identified a greater number of statistically significant temporal clusters in period 1 (1995 to 1999).

**Conclusion:**

These results suggest that human brucellosis persisted annually in Azerbaijan across the study period. The current situation necessitates the development of appropriate surveillance aimed at improving control and mitigation strategies in order to help alleviate the current burden of disease on the population. Areas of concern identified as clusters by the spatial-temporal statistical analyses can provide a starting point for implementing targeted intervention efforts.

## Background

Brucellosis is a widespread zoonotic disease regarded as an emerging and re-emerging threat to public and veterinary health worldwide [1]. Developing nations are often disproportionately afflicted resulting in significant economic losses while at the same time exacting a heavy toll on the health of populations [2,3]. Regions most heavily burdened by the disease include countries of the Mediterranean, Central Asia, Middle East, Latin America, Sub-Saharan African and Balkan Peninsula [4]. The causative agents of the disease are a group of pathogenic bacteria in the genus *Brucella*, which primarily infect animal reservoirs. The primary agents of infection in humans are *B. abortus* (cattle), *B. melitensis* (sheep and goats), *B. suis* (swine), and *B. canis* (dogs) [5]. Humans are often secondarily infected through the consumption of unpasteurized dairy products or coming into contact with infected material during animal husbandry or meat processing [1].

Clinical signs and symptoms of the disease are usually acute and nonspecific, often mimicking other illnesses [6]. Common reported symptoms include fever, malaise, fatigue, sweats, chills, weight loss, and myalgia [7,8]. In some circumstances brucellosis infections can develop into a chronic form, which consists of the continuation of symptoms for greater than twelve months after a diagnosis [7,9]. Despite the reduction or elimination of the disease in many countries through vaccination efforts or increased food safety standards it is estimated that there are nearly 500,000 new cases of the disease each year worldwide [10].

The disease was first diagnosed in Azerbaijan in 1922 and quickly established itself, spreading to more than two thirds of the country’s districts in less than thirty years [11]. Recent governmental changes brought on by the collapse of the Soviet Union in 1991 have likely contributed to the persistence of the disease during the last two decades, due to decreased funding for surveillance and eradication programs [4,12]. Out of the sixty two countries identified as having the highest national incidence of brucellosis, Azerbaijan currently ranks thirteenth with an estimated annual incidence through the year 2000 at over 50 cases per million [4].

Despite the current status of human brucellosis in Azerbaijan, there have been no known published efforts to map and describe the occurrence of the disease at a local level. In order to better understand the spatial and temporal distribution of human brucellosis in Azerbaijan, geospatial analytical techniques and a geographic information system (GIS) were employed. Previous studies using spatio-temporal methods to investigate the distribution of health events have been successful in uncovering factors related to the occurrence of disease [13,14]. Research on brucellosis incorporating spatial analyses identified areas of high human case reporting associated with specific ethnic populations and the consumption of unpasteurized food products in California [6] and Germany [15].

There were three primary objectives of this study: 1) to describe the spatial and temporal distribution of human brucellosis in Azerbaijan; 2) identify the potential presence of clusters of the disease in space and time; and 3) identify epidemiological characteristics of individuals that were diagnosed with an infection.

## Methods

### Ethics statement

No human subjects work was undertaken in this study, human brucellosis case data were extracted from annual government reports. These government reports are prepared public reports, providing summarized count data of patients diagnosed at government health care facilities by category of disease and year. All data were anonymised.

### Data collection and management

Brucellosis is a nationally reportable infectious disease in Azerbaijan. Surveillance and documentation of health events within the country are undertaken by their surveillance and diagnostic laboratory known as the Anti-Plague Station (APS), which is divided into five reporting zones. Each of the five reporting zones has a Regional APS (RAPS) office that responds to health inquiries in order to obtain laboratory samples and verify any diagnosis. In this study a case was defined as any individual with a confirmed positive serology test for *Brucella spp*. Suspected cases of human brucellosis are confirmed by laboratory testing with the Rose Bengal, Huddleson, and the Wright serum agglutination tests. Initial laboratory tests are performed at RAPS, with confirmation (through repeat tests) at the Republican APS.

**Figure 1:**
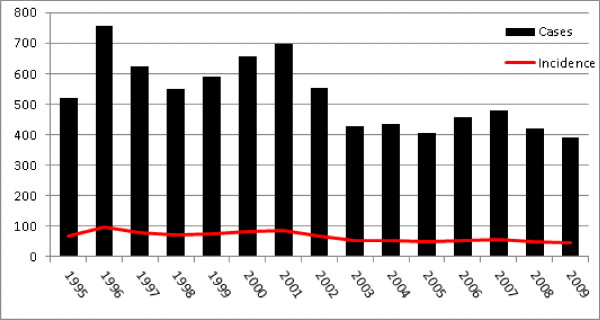
Total number of newly reported cases of human brucellosis seropositives by year during the period 1995 to 2009 shown by the black bars and incidence per 100,000 indicated by the red line.

**Figure 2:**
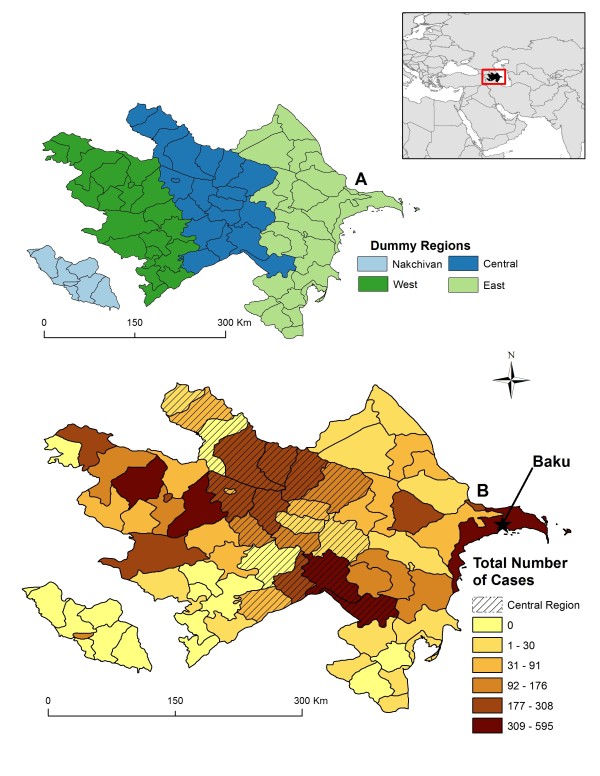
**Map A shows Azerbaijan grouped into dummy regions: Nakchivan (light blue), West (dark green), Central (dark blue), and East (light green).** Map **B** displays the total number of human brucellosis seropositives by district during the time period 1995 to 2009 with the Central region overlain with cross-hatching. The capital Baku is represented by a star in map **B**.

The reporting district was assumed to be the origin of the infection for the individual and the locations of human brucellosis seropositives were aggregated to the 66 districts in Azerbaijan [6]. In order to analyze and describe the spatial and temporal distribution of the disease the 15-year study period was grouped into three, 5-year periods with the total number of new cases per period aggregated to the district level as follows: period 1 (1995 to 1999), period 2 (2000 to 2004), and period 3 (2005 to 2009). Cumulative incidences were calculated per district for each of the three, 5-year periods with the total number of cases during each period as the numerator and the median year population of each 5-year period as the denominator. Population estimates for incidence rates were obtained from the RAPS and the Azeri State Statistical Committee (http://www.azstat.org/). Yearly case totals and the annual incidence of human cases per 100,000 populations in Azerbaijan were recorded during the 15-year study period (Figure 1). Epidemiological data on the age and sex of seropositive cases were obtained from disease registries and stratified by male and female sexes into the following age groups: 0-7, 8-11, 12-15, 16-19, 20-29, 30-39, 40-49, 50-59, and ≥60 years. Azerbaijan was divided into the dummy regions of Nakhchivan, West, Central, and East in order to better describe the spatial and temporal distribution of the disease (Figure 2). It was not possible to match individuals along with their age and gender back to the district level therefore not used in the spatial analytical component of this study. Since brucellosis infections can have symptoms that mimic other diseases and may have an unknown date of onset the date the infection was diagnosed was used in this analysis rather than the date of the onset of symptoms [6].

#### Spatial analysis

Cumulative incidences per 100,000 individuals were mapped at the district level for each of the three 5-year time periods to highlight any spatial changes in risk over time. A second administrative level (district) shapefile of Azerbaijan was obtained from the Global Administrative Areas database (http://www.gadm.org). Smoothed risk estimates were calculated from crude cumulative estimates for each time period using the Empirical Bayesian Smoother (EBS) in the GeoDa software package [16], with the total number of cases in each period as the numerator and the median year population of each period as the denominator. The EBS technique can be used to adjust for instability in the risk estimates caused by heterogeneity in the distribution of cases and the population. It has been suggested that the EBS methodology can be implemented in several scenarios such as when the numerator data total less than three cases, which was the situation in this analysis [17]. In order to maintain a standard comparison of rates between time periods as well as crude and EBS estimates cumulative incidences were choropleth mapped in the following categories per 100,000 persons 0, 0 to 30, >30 to 60, >60 to 90, and >90. All maps were produced in ArcGIS 9.3.1 (ESRI, Redlands, California). Cumulative rates for EBS and crude estimates were compared by dummy region using box plots created in SAS v9.2.

#### Spatial autocorrelation

Local cluster analysis was performed using the Local Moran’s *I* statistic [18], a local indicator of spatial autocorrelation (LISA), in the GeoDa software package [16] using the cumulative incidence rates at the district level in each period as the variable of interest. The statistic can identify hotspots as well as spatial outliers, or in this case individual districts that vary disproportionately from the global mean. Districts are deemed to be not significant or a cluster of either High-High, Low-Low, High-Low, or Low-High values relative to neighboring districts at a given probability level p ≤ 0.05 using 999 randomizations. The null hypothesis states that there is no spatial autocorrelation or association of human brucellosis cases between districts. The local Moran’s *I* statistic is written following [18]:

(1) $$I_{i}=Z_{i}\sum W_{\mathrm{ij}}Z_{j}$$

where I_i_ is the statistic for a district *IZ*_*i*_ is the difference between the brucellosis risk at *I* and the mean brucellosis for Azerbaijan, *Z*_j_ is the difference between brucellosis risk at j and the mean for Azerbaijan. W_ij_ is the weights matrix that in this case only considers neighbors that share a common border or vertex (in the Queen contiguity case W_ij_ is 1/n if a district shares a border or a vertex and zero otherwise).

#### Temporal analysis

Information on the month of diagnosis of the reported cases was examined to identify the presence of a possible seasonality in the reporting of cases. Monthly cases were aggregated into seasons, defined as winter (December, January, February), spring (March, April, May), summer (June, July, August), and fall (September, October, November). Additionally, the Ederer-Myer-Mantel (EMM) test [19] was implemented in ClusterSeer2 [20] in order to explore the presence of temporal clusters of human brucellosis cases by districts in Azerbaijan during the 15-year study period. This test uses the total number of cases per district within a consecutive time period and identifies observations that deviate from an expected number of cases and is written following [19]:

(2) $$x^{2}=\frac{{[|\sum m_{1}-E\left(\sum m_{1}\right)|-0.5]}^{2}}{\sum Var\left(m_{1}\right)}$$

where *m* is the greatest case total for human brucellosis occurring in a district in any of the three time intervals of time series *i.* Since the statistic is biased to changes in population over time [6,19], consecutive time series were limited to 5-year totals resulting in the analysis of three 5-year intervals during the study period. Each of three time intervals for the same district are treated as independent observations in this analysis [6].

## Results

### Spatial analyses

During the study period 1995 to 2009 there were 7,983 reported cases of human brucellosis in Azerbaijan (Figure 1). Annual case totals ranged from 756 in the year 1996 to 392 cases in the year 2009 with a median number of reported cases per year of approximately 522.5 (95% CI: 429, 591). A high percentage of areas reported persistence of disease; out of the 66 districts in the country 65% reported having at least one case of human brucellosis every year during the 15-year study period (Figure 2). The number of districts reporting at least one case for every year in the study also showed regional differences: 1 (16.7%) district in Nakhchivan, 12 (63%) districts in the West, 16 (84%) districts in the Central, and 14 (61%) districts in the East.

**Figure 3:**
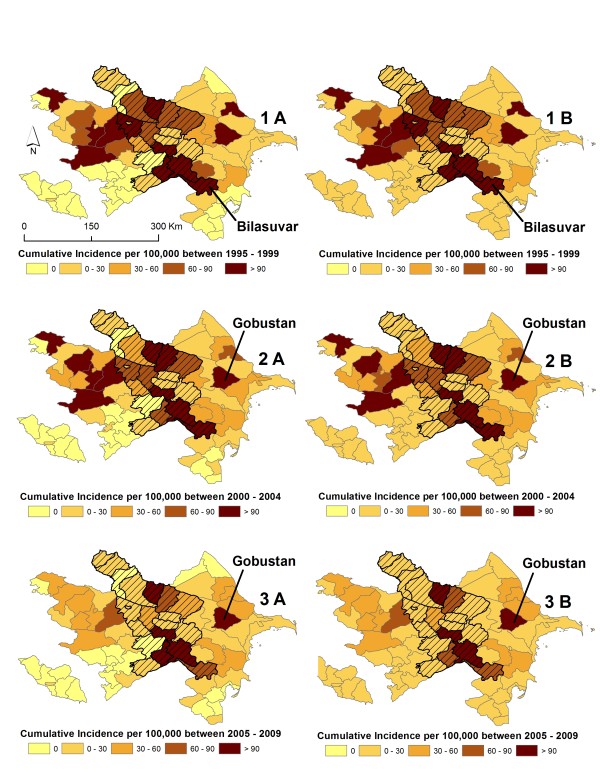
**Spatial distribution of cumulative incidence estimates during the study period 1995 to 2009.** Cumulative estimates were calculated for three equal 5-year periods. The maps display period 1 (1995 to 1999), period 2 (2000 to 2004), and period 3 (2005 to 2009). Insets A refer to crude cumulative incidence estimates for each time period and insets B refer to Empirical Bayes smoothed (EBS) estimates for each time period. Cross-hatching overlain on maps depicts the Central dummy region of Azerbaijan. During period 1 cumulative incidences ranged between 0 to 415.0 cases per 100,000 persons with the highest rate occurring in Bilasuvar (95%CI : 368.9, 465.4), period 2 cumulative incidences that ranged between 0 to 386.5 cases per 100,000 persons with the highest rate occurring in Gobustan (95%CI : 324.5, 456.9), and period 3 Cumulative rates during period 3 ranged between 0 to 283.4 cases per 100,000 persons with the highest incidence again occurring in Gobustan (95%CI : 232.2, 342.8).

**Figure 4:**
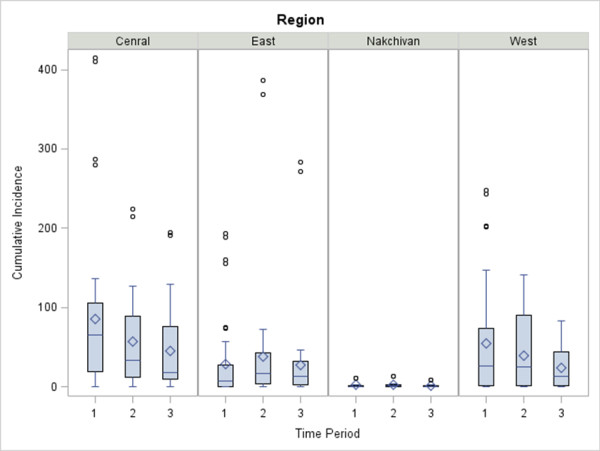
**Boxplots are displaying the crude cumulative incidence rates per district in each of the time periods, [period 1 (1995 to 1999), period 2 (2000 to 2004), and period 3 (2005 to 2009)] paneled by the dummy region: Central, East, Nakchivan, and West.** Crude estimates were calculated for each period using the total number of reported cases during a 5-year period as the numerator and the median year population as the denominator.

Crude and Empirical Bayes Smoothed (EBS) cumulative incidence maps indicated the presence of spatial variation in the distribution of risk across Azerbaijan during the three, 5-year time periods (Figure 3). During period 1 cumulative incidences ranged between 0 to 415.0 cases per 100,000 persons with the highest rate occurring in Bilasuvar (95%CI : 368.9, 465.4) within the Central region. Period 2 displayed cumulative incidences that ranged between 0 to 386.5 cases per 100,000 persons with the highest rate occurring in Gobustan (95%CI : 324.5, 456.9) within the Eastern region. Cumulative rates during period 3 ranged between 0 to 283.4 cases per 100,000 persons with the highest incidence again occurring in Gobustan (95%CI : 232.2, 342.8). EBS cumulative incidence maps illustrated minor variation in the distribution of risk, indicating relative stability in the calculated spatial estimates (Figure 3 see B insets). Boxplots of cumulative rates stratified by time period and method of estimation are shown by region in Figure 4. The distribution of cumulative rates indicated that there was less than 5% variability between the crude and smoothed estimates within regions during each time period.

### Spatial autocorrelation

**Figure 5:**
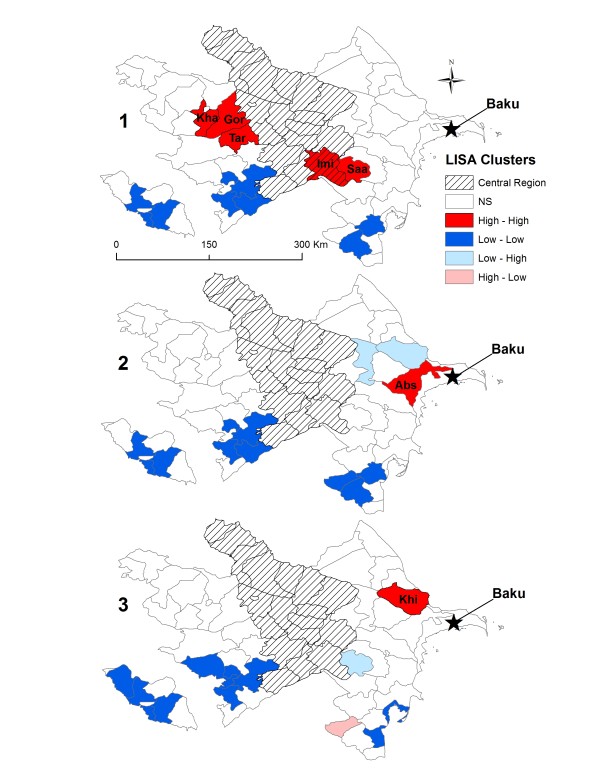
**Local Moran’s *I *clusters across Azerbaijan with red portraying High-High areas, dark blue Low-Low areas, light blue Low-High areas, and pink High-Low areas.** During period 1 (1995 to 1999) High-High clusters were identified in Gor (Goranboy), Imi (Imishli), Kha (Khanlar), Saa (Saatly), and Tar (Tartar). During period 2 (2000 to 2004) Abs (Absheron) was identified as a High-High cluster and Khi (Khizi) was identified during period 3 (2005 to 2009). The star represents the location of the capital Baku and cross-hatching represents the Central region.

The LISA cluster analyses identified significantly high-high clusters of human brucellosis cases at the district level during each of the three periods indicating districts of high incidence surrounded by other districts of high incidence (Figure 5). During period 1 high-high clusters were identified in the west while clusters were found in the east during periods 2 and 3. A total of five high clusters were identified during period 1 that included the districts of Goranboy , Imishli, Khanlar, Saatly, and Tatar with corresponding cumulative rates per 100,000 persons: 203.0 (95% CI; 176.4, 234.8), 101.1 (95% CI: 82.8, 124.3), 116.1 (95% CI: 88.4, 151.9), and 74.2 (95% CI: 55.8, 95.0) respectively. A High-High cluster identified during period 2 included Absheron with 41.1 (95% CI: 28.7, 57.2) cases per 100,000 persons. The LISA analysis for period 3 identified Khizi as a high-high cluster with 36.0 (95% CI: 11.7, 84.0) cases per 100,000 persons. Areas of Low-Low clustering identified by the analyses illustrated an absence or low levels of disease risk among districts in southwestern Azerbaijan that persisted during each time period (Figure 5). A similar pattern held true for the southeastern tip of Azerbaijan with the exception of Yardymli during period 3, which was identified as a High-Low cluster indicating that it had a high cumulative rate compared to neighboring districts.

### Temporal analyses

**Figure 6:**
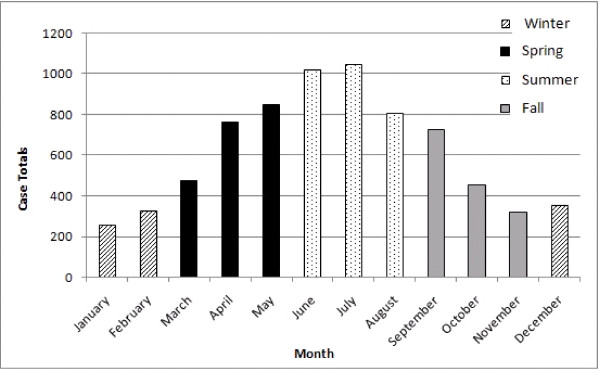
**Total number of human brucellosis cases by month and season.** Winter months (n = 1,000) are shown in the graph by cross-hatched bars, Spring months (n = 2,228) are displayed by the solid black bars, Summer months (n = 3,131) are represented by dotted bars, and Fall months (n = 1,624) are displayed by the solid grey bars.

**Figure 7:**
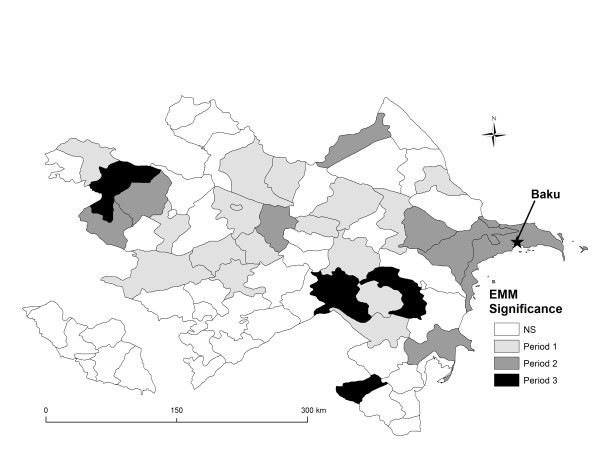
Results from the Ederer-Myer-Mantel’s (EMM) test displaying significant temporal clusters during period 1 (1995 to 1999) shown in light grey, period 2 (2000 to 2004) shown in dark grey, and period 3 (2005 to 2009) shown in black.

There appears to be a distinct seasonality in the temporal distribution of monthly human brucellosis cases (Figure 6). Reporting of cases was highest in the month of July with a total of 1045 cases (13.1%) during the fifteen year period. The Summer season accounted for the greatest number of cases 3,131 (39.2%) followed by Spring 2,228 (27.9%) then Fall 1,624 (20.3%) and lastly Winter with 1,000 cases (12.5%). Results from the EMM test identified 29 districts that were part of statistically significant temporal clusters (Figure 7). There were a greater number of temporal clusters that occurred earlier in the study period; period 1 (n = 16) (χ^2^ = 102.3, p<0.001), followed by period 2 (n = 9) (χ^2^ = 29.5, p<0.001), and period 3 (n = 4) (χ^2^ = 19.2, p<0.01). Temporal clusters from period 1 were located predominantly in the Central and Western regions of Azerbaijan while clusters identified during period 2 and 3 were more evenly dispersed across the country; however, there were no statistically significant human brucellosis clusters identified in the Nakhchivan region.

### Age and gender

**Figure 8:**
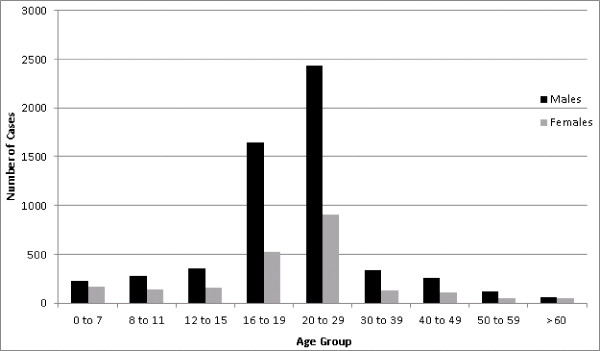
Age distribution of human brucellosis cases in men illustrated by the black bars and women displayed by the grey bars.

Age and sex stratified case totals illustrated a disproportionate number of cases reported among males, with males more heavily burdened by the disease across all age groups (Figure 8). There were a total of 5,730 cases in males accounting for approximately 71.8% of all reported infections. Among age groups for both sexes, 16 to 19 year olds accounted for 2,230 cases [27.9% (95% CI: 27.7, 28.2)] while 20 to 29 year olds accounted for 3,431 cases [43.0% (95% CI: 42.6, 43.3)], with a combined total of 5,661 cases [70.9% (95% CI: 70.6, 71.2)] respectively.

## Discussion

This study represents the first attempts to map and describe local (district level) patterns of human brucellosis in Azerbaijan. We used GIS in conjunction with spatial and temporal analytical methods to analyze patterns of cumulative incidence at the district level in Azerbaijan finding evidence of clustering in both space and time. Additionally, our analyzes confirm that human brucellosis persists annually throughout much of the country, particularly the central region. These current analyses affirm the notion that brucellosis has become endemically established since the first documented reporting in the 1920s.

The results of this study indicated variation in the spatial and temporal distribution of brucellosis reporting in Azerbaijan. Spatial clusters of cumulative incidence were identified in each time period indicating localized areas of high case reporting with clusters predominant in the west early in the study during period 1 and then in the east during periods 2 and 3. Temporal clustering was higher during period 1, but persisted across periods 2 and 3. The median reporting of cases during the study period exceeded 500 cases annually with approximately 65% of districts reporting at least one case every year during the study period, suggesting the possibility of local (district level) persistence. The number of reported cases most likely represents a significant underestimation of the actual disease burden across the country as human brucellosis is often misdiagnosed or is unreported [21]. Additionally, traditional serological tests, like Rose Bengal can have relatively low sensitivity [22]. In Azerbaijan, these issues are likely confounded by the underutilization of the nation’s healthcare facilities [23].

The temporal distribution of cases showed that there was a greater proportion of brucellosis reporting during period 1(1995 to 1999). Temporal clusters identified by the EMM test also indicated a greater number of significant temporal clusters during period 1, which further corroborated that a larger proportion of cases occurring earlier in the study period. Transformations in the funding and organization of public health management and surveillance during the transitional phase to independence have likely contributed to the elevated reporting of the disease in Azerbaijan during this period [23]. However, temporal clustering also suggests that the disease persisted through the more contemporary time period (period 3). The clusters in this most recent period reflect new areas of increased cases reporting that may not be directly attributed to spillover from the Soviet period. This highlights the need for continued surveillance, particularly in those districts identified.

The seasonality of brucellosis can often be attributed to the seasonal birthing of small ruminants (goats and sheep) [24,25]. A recent study in Greece indicated that human cases were directly related to the parturition of small ruminants [26]. In Azerbaijan the birthing of small ruminants occurs in early spring and is often demarcated by an increase in the incidence of livestock brucellosis [11]. Reporting of brucellosis in this study shows a concordance with the spring/summer birthing of small ruminants, with a greater number of cases reported during the summer and spring months (March - August) with a peak in reporting in July. Similar findings related to the seasonality of the disease were also reported in Uzbekistan and Italy [27,28].

As brucellosis is not a contagious disease, spatial clusters of human cases are most likely a result of shared food sources, animal processing, more intensive agricultural production zones, or similar socio-cultural practices [6]. Clusters of disease in this case are also most likely indicative of a larger underlying prevalence of the disease in the local livestock population. High-High districts identified by the LISA analysis during the three time periods indicated a potential shift in the pattern of high case reporting as clusters of the disease present in the west during period 1were absent in periods 2 and 3. Spatial differences in the reporting of brucellosis over time may be a result of changes in agricultural production, with steep declines in production noted during the period 1991 through 1996 followed by efforts to gradually restore sustained agricultural output [29]. These changes have resulted in the subsequent spatial restructuring of agricultural production brought on by the abandonment of government controlled collective farms and animal processing facilities post-collapse of the Soviet Union [30]. Decollectivization marked a shift towards privatization of animals and farms beginning in 1996 [31] resulting in decreased control of veterinary health management policies [22,32]. This has consequently allowed for eased restrictions on the location of agricultural operations as well as trans-boundary movements and production of animals throughout the country [11]. The move towards a more privatized market has fueled expansion around larger cities particularly in the east around the capital Baku [31]. Additionally, the conflict with Armenia, which shares a border with the west of the country has resulted in the displacement of nearly 500,000 Azerbaijani citizens, the occupation of ~20% of Azerbaijan’s land, migration of human and animal populations, and the absence of sanitary-veterinary surveillance; further exacerbating diminished control efforts.

The gender and age distribution of reported cases indicated a higher burden of disease in males (71.8%) compared to females in all age groups. The disproportionate infection rates in males can possibly attributed to differences in the traditional gender roles of men and women in this region. In Azerbaijan, men tend to take on the responsibility of the handling and slaughter of animals in addition to increased occupational hazards such as sheep herding or working in abattoirs [11]. The reporting among age groups showed that individuals age 20 to 29 accounted for the highest percentage of cases (43%) across all age groups and genders, which may reflect on the types of work that are available for individuals in this age group. These finding on the age and gender distribution of cases in Azerbaijan were very similar to those of the neighboring Republic of Georgia [33]. The increased burden of disease in males within the Caucuses is not seen everywhere. For example, in Kampala, Uganda case reporting indicated women represent a higher proportion of brucellosis seropositives [34] whereas in Germany there were equal proportions of reporting between gender groups [15]. Gender differences in case reporting between places may be due to variation in exposures related to occupation or cultural practices such as food preparation. Additionally, there is potential reporting bias between places due to the insidious nature of the disease as well as the utilization and access to healthcare. In Azerbaijan gender differences in the utilization of healthcare facilities may be a source of bias in the reporting of cases. Clark et al. [23] showed that men have a greater tendency not to use healthcare facilities in rural areas when compared to women. However, reporting bias related to occupational exposures are more likely to be biased towards males potentially skewing the case reporting.

The gender and age distribution of cases suggests that food-borne illness may not be the predominant means of transmission for the disease in Azerbaijan. While food-borne illness undoubtedly plays a role in the high incidence of the disease, the male dominated patterns of reporting and skewed age distribution point to high levels of occupational exposure related to transmission of the pathogen. Yet, despite the potential occupational burden of the disease efforts also need to be focused on reducing food-borne transmission as previous research has found that the epidemiology of human brucellosis in some areas can potentially shift away from a disease of those who directly handle animals to a food-borne illness [8]. Several intervention strategies to reduce the burden of brucellosis among human populations have been suggested including: increasing local knowledge of proper food handling techniques of dairy products such as pasteurization [35], decreasing occupational exposures, and vaccination programs aimed at reducing the prevalence of disease in livestock [36]. However, Havaas et al. [37] noted that changing cultural and social behaviors in areas of the Trans-Caucasus region would most likely prove to be difficult and control efforts aimed at vaccinating livestock have been shown to be the most effective [35].

## Limitations

The data presented here were taken from historical records that most likely represent an under reporting of the disease. Analyzing cases obtained from government health care facilities may have introduced biased since individuals in more rural areas may not readily have access to care. Additionally, those individuals in a lower socio-economic strata (SES) may not be able to afford care, medication, or travel to government facilities. These populations within rural areas in a low SES are also more likely to participate in an agricultural related occupation, which may thereby have resulted in underreporting of the burden of disease in this study. Since the onset of brucellosis is often insidious the time of diagnosis was used as the date of infection rather than the onset of the disease. Furthermore, cases obtained from healthcare facilities may not be representative of the general population. This may have skewed the monthly reporting of cases since the incubation period can be greater > 1 month and an individual may not immediately seek care [7]. However, most cases occurred during the spring and summer months and most likely did not dramatically impact cumulative or yearly incidence risk estimates.

There are also limitations to the sensitivity of serological tests for brucellosis and no data were available on the percentage of human cases that were confirmed on bacterial culture, the gold standard for *Brucella* species. Spatial analyses were limited to district level reporting and may not reflect localized areas of infection inside of a given district. The age of individuals also could not be tied back to their district of origin and so no adjustments were made. While it was hypothesized that food was not the main source of infection in Azerbaijan there were limited epidemiological data associated with cases in this study and the data do not provide a definitive link to occupational exposure. Furthermore, the dummy regions created to improve the synthesis of reporting for the results do not represent or delineate national recognizable zones.

## Conclusion

Recent reporting of brucellosis in Azerbaijan indicates a slight decrease in the incidence of disease, yet without adequate livestock surveillance and veterinary health management the brucellosis situation in Azerbaijan may fluctuate widely. The analyses carried out in this study provide a baseline estimation of the spatial and temporal distribution of brucellosis, thereby allowing for the evaluation of deviations from these trends, which could potentially indicate a health emergency in the population. Additionally, the GIS mapping and statistical methodologies may provide a first step in identifying potential problem areas associated with high disease levels. These data can inform policy for implementing appropriate surveillance and control measures. In Azerbaijan where a majority of cases appear to be male dominated as a result of occupational exposures implementing targeted livestock vaccination strategies would seem to be the most prudent choice for control. Districts identified as having high rates of the disease could be initially targeted for directed public health interventions. Future public health management efforts and research in this region should focus on incorporating data on livestock in conjunction with human data to better understand the transmission dynamics of brucellosis.

## Competing interests

The authors declare that they have no competing interests.

## Authors’ contribution

ITK and JKB designed the experiments. RA, RI, NU, AT, ITK constructed the historical GIS data sets. ITK, JKB, RA performed the spatial analysis. ITK performed the spatio-temporal analysis. ITK, JKB, RA, RI drafted the manuscript. All authors read the final draft of the manuscript. RA and ITK contributed equally to this work. All authors read and approved the final manuscript.

## Pre-publication history

The pre-publication history for this paper can be accessed here:

http://www.biomedcentral.com/1471-2334/12/185/prepub
